# The cumulative number of micro-haemorrhages and micro-thromboses in nailfold videocapillaroscopy is a good indicator of disease activity in systemic sclerosis: a validation study of the NEMO score

**DOI:** 10.1186/s13075-017-1354-5

**Published:** 2017-06-13

**Authors:** Romina Andracco, Rosaria Irace, Eleonora Zaccara, Serena Vettori, Wanda Maglione, Antonella Riccardi, Francesca Pignataro, Roberta Ferrara, Domenico Sambataro, Gianluca Sambataro, Claudio Vitali, Gabriele Valentini, Nicoletta Del Papa

**Affiliations:** 1 UOC Day Hospital of Rheumatology, Department of Rheumatology, ASST G. Pini-CTO, Milan, Italy; 2 Rheumatology Unit, Department of Internal Medicine, 2nd University of Naples, Naples, Italy; 3 Section of Rheumatology, Villa San Giuseppe, Istituto Santo Stefano, Como, Italy

**Keywords:** Systemic sclerosis, Nailfold videocapillaroscopy, Disease activity

## Abstract

**Background:**

Some abnormalities in nailfold videocapillaroscopy (NVC), such as the presence of micro-haemorrhages (MHEs), micro-thromboses (MTs), giant capillaries (GCs) and reduction in the number of capillaries (nCs), suggest a disease activity (DA) phase in systemic sclerosis (SSc). In a previous paper, we showed that the number of micro-haemorrhages and micro-thromboses (the so-called NEMO score) was the NVC feature more closely associated with DA. The present study was aimed at validating the NEMO score as a measure of DA in patients with SSc.

**Methods:**

Two cohorts of 122 and 97 patients with SSc who were referred to two different rheumatology units, one in Milan and one in Naples, respectively, constituted the validation cohorts. The NEMO score, the total number of GCs and the mean nCs per digit were the parameters defined in each patient by eight-finger NVC. An expert operator analysed the NVCs in each of the participating units. The European Scleroderma Study Group (ESSG) index was used to define the DA level in each patient at the time of NVC examination.

**Results:**

The NEMO score was the NVC parameter more strictly correlated with the ESSG score in both the Milan and Naples cohorts (*p* < 0.0001), and it was the only one among the NVC variables that gave a significant contribution in a logistic model where the ESSG score represented the dependent variable. ROC curve analysis confirmed that the NEMO score had the best performance in measuring DA. The AUC of the NEMO score was significantly greater than the AUCs obtained by plotting the sensitivity and specificity of the number of GCs and the mean nCs (*p* < 0.0001 in all cases). The NEMO score values that showed the best sensitivity-specificity balance in capturing patients with a relevant DA level were slightly higher in the Naples cohort than in the Milan cohort.

**Conclusions:**

This study confirms that the presence of a certain number of MHEs and MTs in NVC may be considered a strong warning signal of a current phase of DA in patients with SSc.

## Background

Nailfold videocapillaroscopy (NVC) is commonly considered as a feasible method that allows observation and follow-up, in an easily accessible capillary bed, of the micro-vascular changes that characterise the course of systemic sclerosis (SSc) [1]. Some peculiar NVC abnormalities, namely dilated capillaries and avascular areas, have been defined as closely associated with SSc, and consequently these NVC changes have been included as a separate item in the 2013 classification criteria for this disorder [2]. Furthermore, different NVC pictures have been described as potentially predictive of specific disease evolution and some disease-related manifestations [3, 4]. Finally, different NVC aspects have been proposed as being characteristic of the different phases of the disease. Therefore, early, active and late NVC patterns have been carefully described [1]. It has been suggested that the presence of numerous ectasic and giant capillaries (GCs), micro-haemorrhages (MHEs) and micro-thrombosis (MT), and the reduced number of capillaries (nCs) within scattered avascular areas characterise the active pattern [5, 6].

In a previous study, we investigated which of the abnormalities defining the active NVC pattern was more strictly predictive of disease activity (DA) [7]. To do that, we used the proposed and validated European Scleroderma Study Group (ESSG) index as the gold standard for defining DA [8, 9]. We finally demonstrated that the cumulative number of micro-haemorrhages and micro-thromboses (the so-called NEMO score), and to a lesser extent the number of GCs, were positively correlated with the ESSG index [7].

Following this preliminary cross-sectional study that indicates how computing the NEMO score may be a feasible, inexpensive, non-invasive method of measuring the level of DA in patients with SSc, we decided to carry out a second study to validate this new NVC parameter. The present study was performed in two cohorts of patients. The first one was prospectively collected in the centre where the preliminary study was carried out (internal validation cohort), and the second one was retrospectively analysed in a different centre (external validation cohort). The aim of the present study is to confirm the validity of the NEMO score as a measure of DA in SSc when different observers from those who took part in the previous study were asked to measure this NVC parameter. Furthermore, the validity of the NEMO score has been verified by comparing NVC data collected from prospectively enrolled patients in Milan with those derived from the retrospective analysis of stored NVC images of patients from a completely different cohort (Naples).

## Methods

### Patients

The validation cohorts were constituted of a cumulative number of 219 patients. All enrolled patients met the American College of Rheumatology/European League Against Rheumatism (ACR/EULAR) classification criteria for SSc [2], and they were also sub-classified as having limited cutaneous systemic sclerosis (lcSSc) or diffuse cutaneous systemic sclerosis (dcSSc) following the LeRoy et al. criteria [10]. Exclusion criteria were concomitant conditions that may potentially cause additional microvascular changes, such as diabetes, smoking and onychophagic habitus; presence of anti-phospholipid antibodies; and pregnancy. Current treatment with beta-blockers was also an exclusion criterion because it is well known that this drug may cause or exacerbate Raynaud’s phenomenon (RP).

At the time of study enrolment, 52 of the 219 patients were receiving treatment with infusions of intravenous prostanoids (45 with monthly iloprost and 7 with weekly alprostadil), and 16 were taking bosentan. It was decided for ethical reasons to continue the vasodilatory treatments for the prospectively enrolled patients in Milan. Furthermore, all of the patients were receiving stable treatment with low-dose acetylsalicylic acid and calcium channel blockers.

One hundred twenty-two patients with SSc who were referred to the rheumatic disease unit of the Gaetano Pini Institute of Milan formed the prospectively collected internal validation cohort. It was preliminarily established to consecutively include around half the patients with inactive disease (ESSG score <3 and a similar proportion of patients with active disease [ESSG score ≥3]). This cohort did not include any patient who had been recruited for the previous study [7].

Ninety-seven patients with SSc who were referred to the rheumatology unit of the 2nd University of Naples formed the external validation cohort. These patients and their NVC images were randomly selected from the database on the condition that about one-third of them should have an ESSG score ≥3.

### Clinical work-up and assessment of disease activity

The ESSG index was taken as the reference tool (gold standard) for DA assessment. This is a composite scoring system that includes a number of clinical, laboratory and instrument items [8, 9]. According to the ESSG indications, a score ≥3.5 is considered to have good sensitivity and specificity in characterising patients with a high DA level, and a cut-off value of 3.0 [8, 9] also allows capture of patients with a moderate level of DA. The clinical data needed for the ESSG score definition were to be collected within the same month as the NVC examination.

Experienced physicians other than those who performed the NVC were in charge of calculating the modified Rodnan skin score (mRSS). It is well known that the mRSS is a valid and reliable method of assessing skin involvement in SSc and is also closely related to the skin histological features [11–13].

### Nailfold videocapillaroscopy

RA in Milan and RI in Naples were the two investigators responsible for performing and revising the NVC examinations of all the patients in their own centres. Because it is well known that inter-observer reliability is commonly unsatisfactory in medical imaging evaluations, including NVC [14], and because the level of agreement can be improved, even between expert observers, by establishing more precise criteria in pre-study consensus meetings [14, 15], the investigators of the two participating centres entrusted with the task of analysing the NVC images took part in a meeting in Milan before the beginning of the study. During this meeting, these investigators established the precise methodology with which to perform the NVC examination and then performed a pre-test for inter-observer reliability using a series of stored NVC pictures obtained from 30 patients.

The same video capillaroscope with a × 200 magnification lens was used in both centres to examine the nailfold capillaries of all fingers of both hands, excluding thumbs, of each patient. Each digit was positioned in such a way that the capillaroscopic light was 90 degrees incident on the centre of the nailfold. Four consecutive 1-mm fields, for a total extension of 4 mm in the middle of the nailfold, were examined. The derived digital images were stored and analysed using dedicated software (VideoCap; Scalar Co. Ltd., Tokyo, Japan).

For each patient, the following NVC parameters were considered, and the related scores were computed:

1. The NEMO score was calculated by counting the total number of MHEs and MTs observed in the images obtained from the eight fingers of both hands of each patient and defined as the NEMO score. A score of 1 was given to each separate MHE and MT, independently of its size.
2. The GC score was calculated as the total number of GCs found in the eight examined digits. A GC was defined as dilated capillaries with a maximum diameter over 50 μm [16].
3. The nC score was derived by calculating the mean nCs per digit, considering all the NVC fields examined in each finger. The capillary count had to include normal, slightly dilated and ectasic capillaries. The ectasic capillaries were the largely dilated ones, but with a maximum diameter ≤50 μm.

**Fig. 1:**
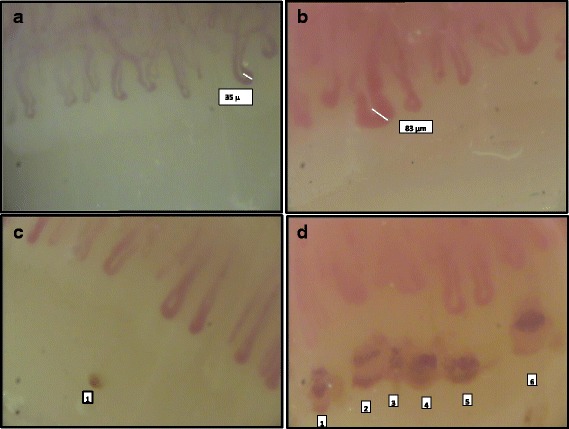
Different nailfold videocapillaroscopic (NVC) features observed in our patients with systemic sclerosis. **a** and **b** A series of dilated capillaries. **a** None of the observed capillaries reaches the limit of 50 μm and so were not defined as giant capillaries (GCs), the larger one having a diameter of 35 μm. **b** One of these capillaries has a diameter of 83 μm, so it can be classified as a GC. **c** An NVC picture where one deposit of hemosiderin (*1*) indicating a previous micro-haemorrhage is present distal to the capillaries aligned in the cuticle. Multiple micro-thromboses are evident in the largely dilated capillaries, and a hemosiderin deposit can be observed distally in the right side of (**d**), just distal to capillary *6*. In (**c**) and (**d**), the number of micro-haemorrhages and micro-thromboses scores were 2 and 49, and the European Scleroderma Study Group index scores were 1 and 7, respectively

Examples of NVC pictures showing an example of a GC and dilated capillaries are shown in Fig. 1a and b. Figure 1c and d show NVC pictures with a high and low number of MHEs and MTs. The respective NEMO and ESSG scores are also reported.

### Statistical methods

Statistical analysis was performed according to standard procedures using IBM SPSS® Statistics version 21 software (IBM, Armonk, NY, USA) and the MedCalc software package, 2014 version (MedCalc® Inc., Ostend, Belgium). Non-parametric tests were used to perform the statistical analysis of the different NVC variables and ESSG scores for DA. This was decided because all the considered NVC variables showed that they did not have a normal distribution (Shapiro-Wilk test, *p* < 0.05 in all the cases). Logistic regression models were built to test the contribution of defined values of NEMO, GC and nC scores in predicting the presence of DA according to the predefined ESSG cut-off value ≥3.

ROC curves were constructed, and the AUC was calculated by plotting the sensitivity and specificity values of the NEMO, GC and nC scores in correctly classifying patients with defined levels of DA. The Hanley-McNeil test was applied to verify the presence of a significant difference between the AUCs obtained by the analysis of different NVC items.

Cohen’s *k* statistics were applied to evaluate the inter-observer level of agreement in the pre-test study on the assessment of NVC abnormalities. No correction of the statistical results was made for the presence of missing values, because there were no missing data in our database. Bonferroni’s correction for multiple comparisons was also applied when indicated.

### Ethical rules

This study was conducted according to the Helsinki declaration and approved by the ethics committee of the Azienda Socio Sanitaria Territoriale Lombardia Centro Specialistico Ortopedico Traumatologico Gaetano Pini of Milan and the 2nd University of Naples, where the study was carried out and where all the study patients included in the two cohorts were recruited. Written informed consent was obtained from all of the enrolled patients.

## Results

The level of agreement between the observers from the two participating centres, which was assessed in the pre-test assay, was good for the NEMO score and number of GCs (*k* = 0.75 and 0.68, respectively) and moderate (*k* = 0.56) when the mean nC value was computed. The internal validation cohort included 122 patients with SSc, of whom 60 and 62 had the limited cutaneous and the diffuse cutaneous variants of SSc, respectively. Ninety-seven patients, of whom 72 and 25 were classified as having lcSSc and dcSSc, respectively, made up the external validation cohort. The main demographic, clinical and serological features of the two populations were separately analysed and compiled and are reported in Table 1. The disease duration appears to be lower in the Milan cohort than in the Naples cohort, but this difference did not reach statistical significance (*p* = 0.06).

**Table 1:** Main demographic data and clinical and laboratory features composing the European Scleroderma Study Group index among patients enrolled in both the internal (Milan) and external (Naples) cohorts

	Milan cohort	Naples cohort	All patients
Number of patients	122	97	219
Male/female ratio	8/114	9/88	17/202
Median age, years (range)	52 (17–82)	55 (19–79)	54 (17–82)
Median disease duration, years (range)	4 (0–28)	6 (0–36)	5 (0–36)
lcSSc/dcSSc	60/62	72/25	132/87
Autoantibodies			
ACAs	57	53	110
Anti-Scl-70	58	22	80
Others	7	22	29
ESSG index ≥3, *n* (%)	57 (46.8)	30 (30.9)	87 (39.7)
ESSG index ≥3.5, *n* (%)	49 (40.2)	20 (20.6)	69 (31.5)
Mean mRSS (range)	5.9 (0–22)	3.4 (0–23)	4.6 (0–23)
Scleroderma, *n* (%)	74 (60.7)	17 (17.5)	91 (41.5)
Change in skin involvement^a^, *n* (%)	35 (28.7)	23 (23.7)	58 (26.4)
Ulcers, *n* (%)	34 (27.9)	11 (11.3)	45 (20.5)
Change in vascular features^a,b^, *n* (%)	56 (46.7)	19 (19.5)	75 (34.2)
Arthritis, *n* (%)	30 (24.6)	7 (7.2)	37 (16.8)
DLCO <80%, *n* (%)	86 (71.3)	85 (87.6)	171 (78)
Change in cardiopulmonary features^a,c^, *n* (%)	25 (20.5)	19 (19.5)	44 (20)
ESR >30 mm/h, *n* (%)	33 (27)	35 (36)	68 (31.1)
Low complement, *n* (%)	15 (12.3)	13 (13.4)	28 (12.7)

*Abbreviations*: *ACAs* Anti-centromere antibodies, *DLCO* Diffusing capacity of the lung for carbon monoxide, *ESR* Erythrocyte sedimentation rate, *ESSG* European Scleroderma Study Group, *dcSSc* Diffuse cutaneous systemic sclerosis, *lcSSc* Limited cutaneous systemic sclerosis, *mRSS* Modified Rodnan skin score

Data are expressed as number of patients (percent), unless otherwise specified

^a^Difference with respect to previous observation

^b^Change in vascular features was defined as subjective feeling of worsening of vascular manifestations (namely Raynaud’s phenomenon)

^c^Change in cardiopulmonary features was defined as subjective feeling of worsening of cardiopulmonary manifestations (namely dyspnoea)

It is worth noting that anti-centromere antibodies (ACAs) were largely predominant in the Naples cohort and that anti-Scl-70 antibodies more frequently found in the Milan cohort. This is a largely expected result when one considers the different prevalence of lcSSc and dcSSc in the two cohorts (Table 1). Of course, the composition of the two cohorts in terms of clinical sub-setting may also be responsible for the different prevalence of some specific clinical features we found in the Milan cohort compared with the Naples cohort (Table 1). Furthermore, these differences can also be ascribed to the fact that patients with active disease (i.e., patients with an ESSG score ≥3) were more prevalent in the Milan cohort than in the Naples cohort (Table 1).

Despite the fact that patients with more active disease and a greater number of patients with dcSSc were present in the Milan cohort, there were no differences in either the ESSG or NEMO scores between the two cohorts (*p* = 0.2 and 0.08, respectively). The distribution of values of the NEMO score was slightly different, however. The highest NEMO score values were recorded in the Milan cohort, whereas values <7 points were found in 40% and 28% of patients in the Milan and Naples cohorts, respectively. Moreover, no differences in the NEMO and ESSG score values were observed, when we considered all the patients of the two cohorts together, between patients with lcSSc and dcSSc with ACAs and anti-Scl-70 antibodies.

In both cohorts, we found that a very strong correlation existed between the NEMO and ESSG scores. A weaker correlation was also present between the ESSG score and the number of GCs in both cohorts, whilst the mean nC score did not correlate with the ESSG score in the Milan cohort and was negatively correlated with the DA score in the Naples cohort (*see* Table 2). Both NEMO and ESSG score values showed a significant negative correlation with disease duration in the Milan cohort (Spearman’s *r* = −0.38 and −0.35, respectively; *p* < 0.0001 in both cases). This kind of correlation was not confirmed in the Naples cohort. The same significant negative correlation between disease duration and both NEMO and ESSG scores was found when all the patients with dcSSc in the two cohorts were pooled together (*r* = −0.39, *p* < 0.0002, and *r* = −0.40, *p* < 0.0001, respectively). The same finding was not confirmed when all the patients of the two cohorts with lcSSc were analysed.

**Table 2:** Correlation between capillaroscopic parameters and European Scleroderma Study Group index score for disease activity

	ESSG score vs NEMO score	ESSG score vs GC count	ESSG score vs capillary count
Milan cohort	*r* = 0.69, *p* < 0.0001	*r* = 0.30, *p* < 0.001	*r* = 0.18, *p* = n.s.
Naples cohort	*r* = 0.76, *p* < 0.0001	*r* = 0.39, *p* < 0.0005	*r* = −038, *p* < 0.0005

*Abbreviations: ESSG* European Scleroderma Study Group index, *GC* Giant capillary, *NEMO* Number of micro-haemorrhages and micro-thromboses, *n.s.* Not significant

*r* = the correlation coefficient by Spearman’s rank correlation. *p* Values were corrected for multiple comparisons by Bonferroni’s method

On the basis of these results, and considering that the aim of our study was simply to validate an NVC scoring system that could predict the presence of a certain degree of DA in all the patients with SSc independently of their clinical subset, we decided to build a logistic regression model in which only the NVC variables (i.e., the NEMO score, CG counts and mean nCs) were considered as independent variables, whereas the presence of an ESSG score ≥3 represented the dependent variable. Only the NEMO score had a significant predictive value for the presence of at least a moderate level of DA in the logistic model constructed for both the Milan and Naples cohorts (*see* Table 3).

**Table 3:** Logistic regression models where number of micro-haemorrhages and micro-thromboses score, giant capillary count and mean number of capillaries were tested as combined predictors of disease activity (European Scleroderma Study Group index score ≥3)

Cohort	Coefficient ± SE	*p* Value	Odds ratio (95% CI)
Milan cohort			
NEMO score	0.36 ± 0.07	<0.0001	1.43 (1.24–1.66)
GC count	−0.002 ± 0.05	0.96	0.99 (0.91–1.09)
Mean number of capillaries	0.14 ± 0 ± 0.01	0.16	1.15 (0.95–1.40)
Constant	−4.19		
Naples cohort			
NEMO score	0.51 ± 0.12	<0.0001	1.67 (1.32–2.11)
GC count	−0.15 ± 0.19	0.45	0.86 (0.59–1.26)
Mean number of capillaries	−0.33 ± 0.22	0.12	0.71 (0.47–1.10)
Constant	−2.90		

*Abbreviations: GC* Giant capillary, *NEMO* Number of micro-haemorrhages and micro-thromboses

**Fig. 2:**
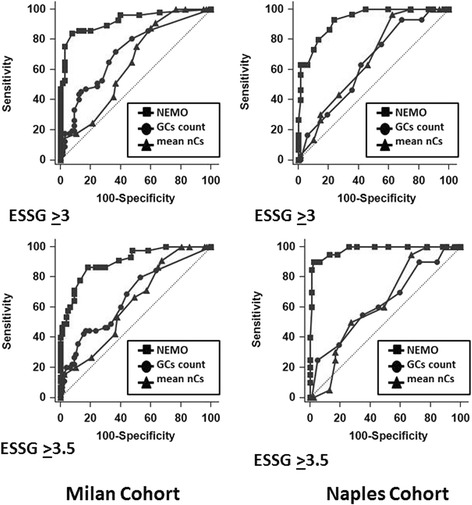
ROC curves built by plotting the sensitivity and specificity values of the number of micro-haemorrhages and micro-thromboses (NEMO) score (*squares*), giant capillary (GC) count (*circles*), and mean number of capillaries (nCs; *triangles*) in correctly classifying patients with either European Scleroderma Study Group (ESSG) index scores ≥3 or ≥3.5 are shown for both the Milan (*left*) and Naples (*right*) cohorts. In both cohorts, the AUC of the NEMO score was always significantly greater than the AUCs designed by plotting both the GC counts and the mean nCs (*p* < 0.0001). Conversely, no differences were found when we compared the AUC of the GC and capillary counts of both cohorts

ROC curves were built by plotting the sensitivity and specificity of the NEMO scores, the number of GCs and mean nCs in correctly classifying patients with either ESSG scores ≥3 or ≥3.5 for both the Milan and Naples cohorts (Fig. 2).

In all cases, the AUC of the NEMO score was significant greater than those designed by plotting both the GC counts and mean nCs (always *p* < 0.0001). Conversely, no differences were found when we compared the AUC of GCs and mean nCs of both cohorts (*see* Fig. 2).

The best performance in terms of sensitivity/specificity ratio in predicting the different levels of DA was obtained in the Milan cohort when the NEMO scores were ≥8 for an ESSG score ≥3. In the Naples cohort, these results differed slightly because the best performance in predicting an ESSG score ≥3 was observed with a NEMO score ≥11 (Table 4). The possibility that these differences could be related to the different ratios of patients with lcSSc and dcSSc in the two cohorts (*see* Table 1) being taken into account. However, there were no differences in the NEMO score between the patients with lcSSc and dcSSc in both cohorts when they were separately analysed and considered as a whole.

**Table 4:** Number of micro-haemorrhages and micro-thromboses scores having best performance in terms of sensitivity/specificity ratio in predicting different degrees of disease activity according European Scleroderma Study Group index score cut-off values

Cohort	Score cut-off values	
Milan cohort	NEMO score ≥8 for ESSG score ≥3	NEMO score ≥9 for ESSG score ≥3.5
	Sensitivity 86.0 (95% CI 74.2–93.7) Specificity 87.7 (95% CI 77.2–94.5)	Sensitivity 86.7 (95% CI 73.2–94.9) Specificity 81.8 (95% CI 71.4–89.7)
Naples cohort	NEMO score ≥11 for ESSG score ≥3	NEMO score ≥15 for ESSG score ≥3.5
	Sensitivity 80.0 (95% CI 61.4–92.3) Specificity 86.6 (95% CI 76.0–93.7)	Sensitivity 90.0 (95% CI 68.3–98.8) Specificity 97.4 (95% CI 90.9–99.7)

*ESSG* European Scleroderma Study Group, *NEMO* Number of micro-haemorrhages and micro-thromboses

## Discussion

In this study, we confirmed that the presence of a given NEMO score is highly indicative of an active phase of SSc. Even the presence of a sufficient number GCs may suggest the presence of DA, although the correlation is stronger for the former NVC abnormality.

The present results may contribute to validating our previous study confirming that a defined level of NEMO score could represent a warning signal that may alert the physician to the possibility that the patient under observation may be in an active phase of SSc. The fact that the correlation between the NEMO score and the ESSG scale also remains strong when the NEMO score is derived by different observers on the basis of stored images and images directly taken of real patients reinforces the validity of this new NVC parameter as a measure of DA. In a previous paper, we also proposed a modified NEMO score based on a logistic regression model where GCs also made a significant contribution to identifying patients with active SSc. The present results did not confirm this, because after constructing the same logistic model in both examined cohorts, only the presence of MHEs and MTs appeared to be effective in capturing patients with active SSc (Table 3). In the previous study, however, the performance in terms of accuracy of the modified NEMO score was not significantly different from that of a simple count of MHEs and MTs (the NEMO score).

The levels of the NEMO score with the best sensitivity/specificity ratio in identifying patients with active disease were slightly different in the two cohorts. However, if one considers this score as a feasible, non-invasive and relatively inexpensive method of suspecting a phase of DA in SSc, this difference actually can be considered marginal. This concept seems to be reinforced by the fact that a NEMO score ≥8 has sensitivity in capturing active patients of 86.0% in the Milan cohort and 96.7% in the Naples cohort, albeit with an expected difference in specificity (87.7% and 69.2% in the Milan and Naples cohorts, respectively).

Besides the known existence of a certain inter-rater variability in NVC evaluation between observers [14], the reasons why the NEMO score demonstrated different performance in the two cohorts in measuring DA remains purely speculative at the moment. This result does not appear to be related to existing differences in the levels of the ESSG score, the prevalence of different disease subsets and their associated serological markers between the cohorts in Milan and Naples. The fact that both ESSG and NEMO scores were closely related to disease duration in the Milan cohort but not in the Naples cohort can be ascribed to the higher prevalence of patients with dcSSc in the Milan cohort. A confirmation of this comes from the evidence that similar negative correlations were also found in the totality of the patients with dcSSc, but not in that of the patients with lcSSc, when the two cohorts were pooled together. The lcSSc and dcSSc variants certainly have differences in their pathological mechanisms, clinical expression and disease course. It is well known dcSSc can be a more aggressive condition where active phases are commonly more concentrated in the early phase of the disease [10, 17]. However, to our knowledge, no specific longitudinal studies have been performed so far comparing NVC pictures and their evolution over time in two different subsets of SSc.

Some studies have shown that the clinical expression of SSc may vary in different geographical areas [18, 19]. RP, including RP related to SSc, may have a different presentation pattern in terms of the number and severity of episodes in relation to seasonal changes and local climate [18–20]. There are no specific surveys demonstrating that the NVC patterns may change in populations that differ because of ethnic origin or geographic localization. However, climate is certainly different in Milan and Naples, particularly for the lower temperatures in winter in Milan, which has a Continental climate, and the longer summer season, with usually higher temperatures, that characterises Naples, around 800 kilometres farther south. How these climatic differences may influence the NVC pattern and the natural course of SSc in the patients referred to the two rheumatologic units in the present study is a completely unknown issue at present.

NVC is a feasible technique widely used in the diagnostic approach [1] and in disease monitoring for patients with SSc [4]. A relevant prognostic value has also been ascribed to NVC, and different NVC patterns have been defined as being predictive of a worse outcome [21]. Finally, different NVC features have been proposed as characteristic of the early, active and late phases of disease [1, 6]. In particular, the features that define the active disease phase are the presence of MHEs and GCs together with an initial loss of capillaries.

It is not absolutely surprising that MHEs and MTs were the NVC abnormalities more closely related to DA when one considers the present knowledge on the small vessel vasculopathy of SSc. SSc is commonly defined as a disease of the micro-vascular bed of the skin compartment, but potentially involving the small vessels of internal organs. The endothelial involvement is believed to be the first step of the pathological process in SSc, whereas homing of mononuclear inflammatory cells in the interstitium, activation of myofibroblast and fibroblast lineage, together with the consequent fibrotic changes, could be considered as the following evolutionary changes [22, 23]. The typical response to the initial micro-vascular damage and capillary loss in SSc is the compensatory dilation of remaining capillary loops, sometimes with the appearance of formation of GCs. In late disease, enlarged capillaries are much less frequently found, whereas an evident capillary loss and the appearance of vascular areas are the most typical NVC features.

In progressive disease, the destiny of enlarged capillaries is thrombotic obliteration followed by extravasation [24, 25]. When these phenomena are synchronous, several extravasations with hemosiderin deposits aligned in the cuticle distal from capillary areas can be observed by NVC.

Koenig et al. [26] described the appearance of dilated capillaries as an early event, whereas loss of capillaries and telangiectases were observed later. This is partially in contrast with our findings, suggesting that GCs may appear early together with the other enlarged capillaries. However, it has been reported that the observation of enormous GCs in the late phase of the disorder is not a rare finding [25]. This NVC feature may correspond to the stable telangiectases that can often be observed in the skin of patients with late SSc. Of course, some differences between Koenig’s survey and the present study can be largely explained by the different structure of the two studies. The first one was a longitudinal study aimed at investigating NVC changes during the evolution from isolated RP to overt SSc [26]. The present one is a cross-sectional study aimed at understanding which of the NVC features can be more indicative of an active phase of the disease in patients with already defined SSc. Therefore, the terms *early* and *late* may not have equal meaning because of the differences in the design of the two studies.

The finding that the presence of a given amount of MHEs and MTs (more closely than that of GCs) predicts that the DA phases in SSc may simply indicate that these abnormalities could represent the NVC aspects corresponding to the evolution of microvascular involvement in the active phases of the disease. It is self-evident that DA in SSc is characterised and conditioned by a large number of other, more complex and still poorly understood pathological mechanisms [21, 22].

The present study confirms that the NEMO score can be considered a feasible and valid method of identifying patients with SSc who may cross a phase of DA. Of course, this kind of suspicion should be confirmed by an accurate clinical and instrumental work-up of the observed patient. Nevertheless, NEMO score assessment might help the clinician in measuring DA when other items are not available. In this respect, a longitudinal study where the NEMO score and level of DA may be contemporaneously measured along the disease course in a large series of patients is certainly needed to definitively confirm the utility of such a simple NVC scoring system, not only as a steady-state scoring system of DA but also as a reliable transition index to be applied in the follow-up of patients with SSc.

The comparison between the internal and external validation cohorts examined in the study may suggest that the NVC abnormalities could be influenced by some environmental factors, and consequently the NEMO score levels that can identify active patients should be verified at all centres. EULAR Scleroderma Trials and Research Group investigators recently identified and validated a new activity index that performs better than the previous ESSG activity index used in this study and in the previous one [27]. A study has just been set up in our centres to investigate the relationships between the NEMO score and the revised activity index.

## Conclusions

The present study confirms that the presence of a certain NEMO score in NVC may be considered as an indirect measure of a current phase of DA in patients with SSc. Even the presence of a sufficient number GCs may suggest the presence of DA, although the correlation is stronger for the former NVC abnormality. These results contribute to validating our previous study by demonstrating that the presence of a defined number of MHs and MTs (the so-called NEMO score) could represent a warning signal that may alert the physician to the possibility that the patient under observation may be in an active phase of SSc.

## Abbreviations

ACA: Anti-centromere antibodies
ACR/EULAR: American College of Rheumatology/European League Against Rheumatism
DA: Disease activity
dcSSc: Diffuse cutaneous systemic sclerosis
DLCO: Diffusing capacity of the lung for carbon monoxide
ESR: Erythrocyte sedimentation rate
ESSG: European Scleroderma Study Group
GC: Giant capillary
lcSSc: Limited cutaneous systemic sclerosis
MHE: Micro-haemorrhage
mRSS: Modified Rodnan skin score
MT: Micro-thrombosis
nC: Number of capillaries
NEMO: Number of micro-haemorrhages and micro-thromboses
NVC: Nailfold videocapillaroscopy
RP: Raynaud’s phenomenon
SSc: Systemic sclerosis
